# Comparative study of the short-term outcomes of gastric cancer surgery between Japanese Gastric cancer association-certified and non-certified institutions: a retrospective cohort analysis using a national database in Japan

**DOI:** 10.1007/s10120-025-01694-8

**Published:** 2026-01-02

**Authors:** Tomoyuki Matsunaga, Hideki Endo, Hiroyuki Yamamoto, Koshi Kumagai, Shingo Kanaji, Hisato Kawakami, Chika Kusano, Ryoji Kushima, Mitsuhiro Fujishiro, Kensei Yamaguchi, Takaki Yoshikawa, Yuichiro Doki, Yoshihiro Kakeji, Yoshiyuki Fujiwara

**Affiliations:** 1 Division of Gastrointestinal and Pediatric Surgery, Department of Surgery, School of Medicine, Faculty of Medicine, Tottori University, 36-1 Nishi-Cho, Yonago, 683-8504 Japan; 2 Department of Healthcare Quality Assessment, Graduate School of Medicine, The University of Tokyo, Tokyo, Japan; 3 Department of Upper Gastrointestinal Surgery, Kitasato University School of Medicine, 1-15-1 Kitasato, Minami-Ku, Sagamihara, Kanagawa 252-0374 Japan; 4 Division of Gastrointestinal Surgery, Department of Surgery, Kobe University Graduate School of Medicine, 7-5-2 Kusunoki-Cho, Chuo-Ku, Kobe, Japan; 5 Department of Clinical Oncology, Tohoku University Graduate School of Medicine, Sendai, Japan; 6 Department of Gastroenterology, Kitasato University School of Medicine, Kanagawa, 252-0374 Japan; 7 Department of Pathology, Shiga University of Medical Science, Otsu, Japan; 8 Department of Gastroenterology, Graduate School of Medicine, The University of Tokyo, Tokyo, Japan; 9 Department of Gastroenterological Chemotherapy, Cancer Institute Hospital of the Japanese Foundation for Cancer Research, Tokyo, Japan; 10 Department of Gastric Surgery, National Cancer Center Hospital, Chuo-Ku, Tokyo, Japan; 11 Department of Gastroenterological Surgery, Osaka University Graduate School of Medicine, Osaka, Japan

**Keywords:** Certified institution, Clinical database, Gastric cancer, Postoperative complication, Short-term outcome

## Abstract

**Background:**

This study assessed the impact of an institutional certification system that was newly introduced by the Japanese Gastric Cancer Association on short-term surgical outcomes in patients with gastric cancer using data from the National Clinical Database.

**Methods:**

A retrospective cohort study of distal gastrectomy and total gastrectomy procedures performed between January 2020 and December 2022 was conducted. The institutions were classified into three categories: type A, type B, and non-certified institutions, in decreasing order of certification stringency. The primary outcome was the incidence of grade ≥ IIIa postoperative complications based on the Clavien–Dindo classification system. The secondary outcome was surgery-related mortality. Logistic regression with risk adjustment, estimated using generalized estimating equations, was used to account for intra-cluster correlation.

**Results:**

There was no significant difference in the risks of distal gastrectomy-related complications across the three institution types. However, type A- (odds ratio (OR) 0.39, 95% confidence interval (CI) 0.31–0.49) and type B-certified institutions (OR 0.59, 95% CI 0.49–0.71) had a significantly lower mortality risk than non-certified ones. On the other hand, Type A- (OR 1.25, 95% CI 1.09–1.44) and type B-certified institutions (OR 1.17, 95% CI 1.03–1.33) had higher risks of postoperative total gastrectomy-related complications than non-certified ones. Nevertheless, type A- (OR 0.41, 95% CI 0.29–0.58) and type B-certified institutions (OR 0.67, 95% CI 0.51–0.88) had significantly lower surgery-related mortality risks than non-certified ones.

**Conclusions:**

Certified institutions demonstrated lower surgical mortality risks, highlighting the benefits of the certification system and the importance of institutional quality.

## Introduction

Gastric cancer is the fifth leading cause of cancer-related mortality worldwide [1]. Although its incidence has decreased due to the widespread use of *Helicobacter pylori* eradication treatment, the patient population is aging and exhibiting higher-risk profiles [2, 3]. In recent years, gastric cancer surgery has rapidly advanced toward less invasiveness with the development of laparoscopic and robot-assisted surgeries, resulting in the need for higher levels of surgical technique and specialized postoperative management [4, 5]. Improvement in short-term postoperative outcomes is closely related to long-term prognosis and quality of life. Therefore, it is important to evaluate the skill level and system of each institution [6, 7]. In fact, previous nationwide studies utilizing the National Clinical Database (NCD) have shown that high-volume centers can achieve better short-term surgical outcomes for gastric cancer compared with low-volume institutions [8, 9]. The Japanese Society for Esophageal Cancer Research has established specialist and certified facility systems and reported that these facilities have achieved good surgical treatment outcomes [10].

In response to these evolving circumstances, the Japanese Gastric Cancer Association (JGCA) introduced an institutional certification system in 2023. The accreditation criteria include not only a specific number of surgical procedures but also the use of endoscopic treatments and chemotherapy (Supplemental Table 1). In addition, the presence of several board-certified oncologists and collaboration among different departments, which can provide comprehensive gastric cancer care treatment, are required. Under this system, certified institutions are categorized into types A and B based on specific application criteria. Type A institutions can offer highly advanced gastric cancer treatment. Meanwhile, type B institutions have slightly less stringent standards but can still provide high-quality care. However, the efficacy and impact of this accreditation system are yet to be fully evaluated.

**Table 1:** Preoperative, intraoperative, and postoperative characteristics of patients undergoing distal gastrectomy stratified by institutional certification status

	All institutions (n = 1917)	Non-certified institutions (n = 1471)	Type B-certified institutions (n = 298)	Type A-certified institutions (n = 148)
Total number of surgical cases	74,311	31,656	21,193	21,462
Preoperative factors				
Age ≥ 70 years	50,518 (68.0)	22,637 (71.5)	14,464 (68.2)	13,417 (62.5)
Female sex	24,714 (33.3)	10,380 (32.8)	6871 (32.4)	7463 (34.8)
BMI				
≥ 18.5, < 25 kg/m^2^	48,927 (65.8)	20,755 (65.6)	13,898 (65.6)	14,274 (66.5)
< 18.5 kg/m^2^	9110 (12.3)	4292 (13.6)	2536 (12.0)	2282 (10.6)
≥ 25 kg/m^2^	16,274 (21.9)	6609 (20.9)	4759 (22.5)	4906 (22.9)
Diabetes mellitus	15,727 (21.2)	6849 (21.6)	4589 (21.7)	4289 (20.0)
Smoking	38,998 (52.5)	15,159 (47.9)	11,710 (55.3)	12,129 (56.5)
Dependence in activities of daily living	3881 (5.2)	2162 (6.8)	1050 (5.0)	669 (3.1)
Chronic obstructive pulmonary disease	3784 (5.1)	1352 (4.3)	1034 (4.9)	1398 (6.5)
Dialysis	684 (0.9)	295 (0.9)	218 (1.0)	171 (0.8)
History of ischemic heart disease	3382 (4.6)	1460 (4.6)	1073 (5.1)	849 (4.0)
Congestive heart failure (within 30 days)	603 (0.8)	336 (1.1)	162 (0.8)	105 (0.5)
Long-term steroid use	828 (1.1)	326 (1.0)	224 (1.1)	278 (1.3)
Weight loss	2863 (3.9)	1500 (4.7)	737 (3.5)	626 (2.9)
Preoperative blood transfusion	1941 (2.6)	1170 (3.7)	490 (2.3)	281 (1.3)
Hemoglobin level < 13.5 g/dL in men, < 11.5 g/dL in women	24,143 (32.5)	10,511 (33.2)	6888 (32.5)	6744 (31.4)
Albumin level < 3.5 g/dL	14,721 (19.8)	7495 (23.7)	4167 (19.7)	3059 (14.3)
Blood urea nitrogen level < 8 mg/dL	1518 (2.0)	728 (2.3)	451 (2.1)	339 (1.6)
Creatinine level > 1.2 mg/dL	7117 (9.6)	3206 (10.1)	2108 (9.9)	1803 (8.4)
Aspartate aminotransferase level > 35 IU/L	5414 (7.3)	2375 (7.5)	1562 (7.4)	1477 (6.9)
Preoperative chemotherapy	2649 (3.6)	855 (2.7)	761 (3.6)	1033 (4.8)
Preoperative radiotherapy	88 (0.1)	21 (0.1)	26 (0.1)	41 (0.2)
≥ T3 (TNM classification)	28,436 (38.3)	13,067 (41.3)	8332 (39.3)	7037 (32.8)
≥ N1 (TNM classification)	27,321 (36.8)	12,553 (39.7)	8109 (38.3)	6659 (31.0)
M1 (TNM classification)	3339 (4.5)	1588 (5.0)	1012 (4.8)	739 (3.4)
ASA-PS score of 3–5	12,196 (16.4)	5580 (17.6)	3661 (17.3)	2955 (13.8)
Intraoperative characteristics				
Median (IQR) surgical duration, min	274 [219–337]	258 [202–323]	280 [227–340]	288 [236–351]
Median (IQR) estimated blood loss volume, mL	50 [10–160]	80 [20–216]	40 [9–150]	21 [5–89]
Surgical approach (laparoscopic or robotic)	50,204 (67.6)	17,045 (53.8)	15,256 (72.0)	17,903 (83.4)
R1, R2 resection	3558 (4.8)	1773 (5.6)	1006 (4.7)	779 (3.6)
Outcome				
Postoperative complication (CD3)	4680 (6.3)	2095 (6.6)	1359 (6.4)	1226 (5.7)
Surgery-related mortality	759 (1.0)	494 (1.6)	175 (0.8)	90 (0.4)

Data were presented as the number (percentage) of patients, if not otherwise stated

ASA-PS: American Society of Anesthesiologists Physical Status, BMI: body mass index, CD: Clavien–Dindo classification, IQR: interquartile range

In the current study, we analyzed data from the NCD to compare short-term outcomes after surgery between certified institutions (types A and B) and non-certified institutions. This comparison is expected to offer some objective insights into the relevance of the JGCA certification system.

## Methods

### Data collection

This retrospective cohort study used data registered in the NCD, which is a nationwide registry established in 2010 to improve the quality of surgical care and clinical outcomes in Japan [11]. The NCD is a comprehensive web-based database that collects data on clinical information and surgical outcomes from over 5,000 participating institutions, covering > 95% of surgical procedures performed in Japan [12]. Further, it obtains data on all types of gastroenterological surgeries and evaluates the quality of gastric cancer surgeries based on detailed information from the preoperative, intraoperative, and postoperative periods. In the NCD data entry system, missing values are not permitted except in cases where the tests were not performed. Moreover, random audits are conducted at participating institutions to validate data consistency. The database, which is managed by the Japan Surgical Society and linked to the board certification system for surgeons, ensures a high level of data accuracy and completeness. The NCD is widely used for clinical research and quality improvement initiatives. Thus, it is a reliable source of real-world data for observational studies.

### Patients

Patients diagnosed with gastric cancer who underwent distal gastrectomy and total gastrectomy between January 2020 and December 2022 were included in this analysis. This period was set because the procedures performed during this time were used for the certified institutions. The exclusion criteria were as follows: patients aged < 18 years, those who underwent emergency surgery, those with missing data on laboratory blood test results or surgical outcomes, and those who underwent combined resection of organs other than the gallbladder, spleen, or ovaries.

### Endpoint

Comparative analyses were conducted among the aforementioned type A-certified institutions, type B-certified institutions, and non-certified institutions. The primary outcome measure was the incidence of grade ≥ IIIa postoperative complications, as defined by the Clavien–Dindo classification system. This system, proposed by Dindo et al., is used to assess the severity of postoperative complications and to facilitate comparisons among different hospitals [13]. Grade ≥ IIIa complications require surgical, endoscopic, or radiologic intervention. The secondary outcomes included surgery-related mortality, defined as all deaths occurring within 30 days after surgery (including post-discharge) and during the index hospitalization period. This definition has been widely used in previous studies utilizing the NCD.

### Statistical analysis

To examine the association between institutional certification status and postoperative outcomes, separate logistic regression models for distal gastrectomy and total gastrectomy were established. These models were utilized to evaluate the association of postoperative complications and surgery-related mortality with facility type—namely, type A-certified institutions, type B-certified institutions, and non-certified institutions (reference group). Generalized estimation equations were used to account for the potential clustering of patients within hospitals.

For risk adjustment in this study, based on previous publications, we focused on items specific to gastrointestinal surgery within the NCD [14]. The covariates included the demographic characteristics of the participants, comorbidities, and preoperative laboratory values. These were as follows: age (< 70 vs ≥ 70 years); sex (male vs female); body mass index (BMI) (< 18.5 vs ≥ 18.5, < 25 vs ≥ 25 kg/m^2^); presence of diabetes mellitus; smoking history; functional independence in activities of daily living; history of chronic obstructive pulmonary disease; history of dialysis; ischemic heart disease; congestive heart failure; long-term corticosteroid use; weight loss of > 10%; history of preoperative blood transfusion; hemoglobin level (male: < 13.5 vs ≥ 13.5 g/dL; female: < 11.5 vs ≥ 11.5 g/dL); serum albumin level (< 3.5 vs ≥ 3.5 g/dL); blood urea nitrogen level (< 8.0 vs ≥ 8.0 mg/dL); creatinine level (< 1.2 vs ≥ 1.2 mg/dL); aspartate aminotransferase level (< 35 vs ≥ 35 IU/L); treatment with chemotherapy or radiotherapy; clinical T factor (T3–4 vs T0, Tis, T1a, T1b, T2, and Tx), N factor (N1–3 vs N0, Nx), and M factor (0 vs 1) according to the 7th edition of the Union for International Cancer Control Tumor-Node-Metastasis classification; and American Society of Anesthesiologists Physical Status classification score (1–2 vs ≥ 3). All P values were two-sided, and a P value of < 0.05 indicated statistically significant differences. All statistical analyses were conducted with R version 4.4.1 (2024; R Foundation for Statistical Computing, Vienna, Austria).

## Results

### Characteristics of patients who underwent distal gastrectomy

**Fig. 1:**
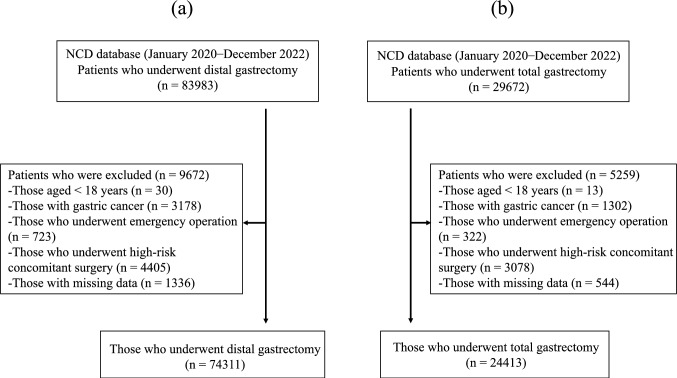
Patient flow diagram. **a** Distal gastrectomy. **b** Total gastrectomy. NCD, National clinical database

In the current study, 83,983 cases of distal gastrectomy were collected from 1,917 institutions in the NCD. After applying the exclusion criteria, 74,311 cases were analyzed (Fig. 1a). Table 1 shows the results. In total, 1,471 non-certified institutions, 298 type B-certified institutions, and 148 type A-certified institutions accounted for 31,656, 21,193, and 21,462 cases, respectively.

In terms of preoperative characteristics, non-certified institutions treated a higher proportion of elderly patients aged ≥ 70 years (71.5%, 68.2%, and 62.5%, respectively), those with a low BMI (< 18.5 kg/m^2^; 13.6%, 12.0%, and 10.6%, respectively), those with low serum albumin levels (< 3.5 g/dL; 23.7%, 19.7%, and 14.3%, respectively), and those requiring preoperative blood transfusion (3.7%, 2.3%, and 1.3%, respectively) than type B- and type A-certified institutions. In contrast, preoperative chemotherapy was more common in type A-certified institutions (4.8%) than in type B-certified institutions (3.6%) and non-certified centers (2.7%).

Regarding intraoperative findings, patients in non-certified institutions (258 min) had a shorter median surgical duration than those in type B-certified institutions (280 min) and type A-certified institutions (288 min). Meanwhile, patients in non-certified institutions had a greater estimated blood loss volume than those in type B- and type A-certified institutions (80, 40, and 21 mL, respectively). The use of minimally invasive surgery (MIS) was lower in patients in non-certified institutions (53.8%) than in those in type B-certified (72.0%) and type A-certified (83.4%) institutions.

### Outcomes of patients who underwent distal gastrectomy

In terms of postoperative outcomes, the incidence rate of grade ≥ 3 complications based on the Clavien–Dindo classification system was slightly higher in non-certified centers (6.6%) than in type B-certified (6.4%) and type A-certified (5.7%) institutions. However, non-certified institutions (1.6%) had the highest surgery-related mortality rate, followed by type B-certified (0.8%) and type A-certified (0.4%) institutions.

When evaluating the association between postoperative complications and surgery-related mortality and facility type after adjusting for the aforementioned covariates, the risk of postoperative complications did not significantly differ between type B-certified institutions (risk-adjusted odds ratio [OR]: 0.99, 95% confidence interval [CI]: 0.89–1.10) and type A-certified institutions (risk-adjusted OR: 0.97, 95% CI: 0.88–1.08) compared with non-certified institutions. However, type B-certified institutions (risk-adjusted OR: 0.59, 95% CI: 0.49–0.71) and type A-certified institutions (risk-adjusted OR: 0.39, 95% CI: 0.31–0.49) had a significantly lower postoperative mortality risk than non-certified institutions (Table 2).

**Table 2:** Association between institutional certification status and (a) postoperative complications and (b) surgery-related mortality after distal gastrectomy

(a)			
Facility-certified attributes	Unadjusted odds ratio (95% CI)	Risk adjusted odds ratio (95% CI)	P value
Non-certified institutions	References	References	
Type B-certified institutions	0.97 (0.90–1.04)	0.99 (0.89–1.10)	0.81
Type A-certified institutions	0.85 (0.79–0.92)	0.97 (0.88–1.08)	0.62

(b)			
Facility-certified attributes	Unadjusted odds ratio (95% CI)	Risk adjusted odds ratio (95% CI)	P value
Non-certified institutions	Reference	Reference	
Type B-certified institutions	0.53 (0.44–0.62)	0.59 (0.49–0.71)	< 0.001
Type A-certified institutions	0.27 (0.21–0.33)	0.39 (0.31–0.49)	< 0.001

CI: confidence interval

### Characteristics of patients who underwent total gastrectomy

In total, 29,672 cases of total gastrectomy were collected from 1,732 institutions in the NCD. After applying the exclusion criteria, 24,413 cases were analyzed (Fig. 1b).

Table 3 shows the results. A total of 1,287 non-certified institutions, 297 type B-certified institutions, and 148 type A-certified institutions accounted for 11,674, 6,575, and 6,164 cases, respectively.

**Table 3:** Preoperative, intraoperative, and postoperative characteristics of patients undergoing total gastrectomy stratified by institutional certification status

	All institutions	Non-certified institutions	Type B-certified institutions	Type A-certified institutions
	(n = 1732)	(n = 1287)	(n = 297)	(n = 148)
Total number of surgical cases	24,413	11,674	6575	6164
Preoperative factors				
Age ≥ 70 years	16,537 (67.7)	8189 (70.1)	4491 (68.3)	3857 (62.6)
Female sex	6319 (25.9)	2914 (25.0)	1712 (26.0)	1693 (27.5)
BMI				
≥ 18.5, < 25 kg/m^2^	16,151 (66.2)	7640 (65.4)	4344 (66.1)	4167 (67.6)
< 18.5 kg/m^2^	3646 (14.9)	1870 (16.0)	947 (14.4)	829 (13.4)
≥ 25 kg/m^2^	4616 (18.9)	2164 (18.5)	1284 (19.5)	1168 (18.9)
Diabetes mellitus	5050 (20.7)	2526 (21.6)	1338 (20.3)	1186 (19.2)
Smoking	13,790 (56.5)	6166 (52.8)	3886 (59.1)	3738 (60.6)
Dependence in activities of daily living	1054 (4.3)	609 (5.2)	283 (4.3)	162 (2.6)
Chronic obstructive pulmonary disease	1317 (5.4)	568 (4.9)	307 (4.7)	442 (7.2)
Dialysis	158 (0.6)	92 (0.8)	35 (0.5)	31 (0.5)
History of ischemic heart disease	1040 (4.3)	496 (4.2)	303 (4.6)	241 (3.9)
Congestive heart failure (within 30 days)	165 (0.7)	103 (0.9)	40 (0.6)	22 (0.4)
Long-term steroid use	228 (0.9)	105 (0.9)	64 (1.0)	59 (1.0)
Weight loss	1578 (6.5)	821 (7.0)	431 (6.6)	326 (5.3)
Preoperative blood transfusion	782 (3.2)	483 (4.1)	190 (2.9)	109 (1.8)
Hemoglobin level < 13.5 g/dL in men, < 11.5 g/dL in women	9838 (40.3)	4671 (40.0)	2598 (39.5)	2569 (41.7)
Albumin level < 3.5 g/dL	6038 (24.7)	3105 (26.6)	1647 (25.0)	1286 (20.9)
Blood urea nitrogen level < 8 mg/dL	623 (2.6)	310 (2.7)	168 (2.6)	145 (2.4)
Creatinine level > 1.2 mg/dL	2210 (9.1)	1143 (9.8)	596 (9.1)	471 (7.6)
Aspartate aminotransferase level > 35 IU/L	2047 (8.4)	934 (8.0)	548 (8.3)	565 (9.2)
Preoperative chemotherapy	2289 (9.4)	767 (6.6)	609 (9.3)	913 (14.8)
Preoperative radiotherapy	30 (0.1)	8 (0.1)	8 (0.1)	14 (0.2)
≥ T3 (TNM classification)	15,784 (64.7)	7390 (63.3)	4362 (66.3)	4032 (65.4)
≥ N1 (TNM classification)	13,433 (55.0)	6456 (55.3)	3734 (56.8)	3243 (52.6)
M1 (TNM classification)	2350 (9.6)	1163 (10.0)	652 (9.9)	535 (8.7)
ASA-PS score of 3–5	3916 (16.0)	2047 (17.5)	1060 (16.1)	809 (13.1)
Intraoperative characteristics				
Median (IQR) surgical duration, min	300 [235–379]	277 [218–353]	307 [245–382]	337 [268–420]
Median (IQR) estimated blood loss volume, mL	176 [50–400]	206 [80–448]	153 [50–376.5]	130 [30–334]
Surgical approach (laparoscopic or robotic)	9801 (40.1)	3405 (29.2)	2954 (44.9)	3442 (55.8)
R1, R2 resection	2412 (9.9)	1204 (10.3)	688 (10.5)	520 (8.4)
Outcome				
Postoperative complication (CD3)	2401 (9.8)	1084 (9.3)	681 (10.4)	636 (10.3)
Surgery-related mortality	393 (1.6)	261 (2.2)	88 (1.3)	44 (0.7)

Data were presented as the number (percentage) of patients, if not otherwise stated

ASA-PS: American Society of Anesthesiologists Physical Status, BMI: body mass index, CD: Clavien–Dindo classification, IQR: interquartile range

In terms of preoperative characteristics, non-certified institutions treated a higher proportion of elderly patients aged ≥ 70 years (70.1%, 68.3%, and 62.6%, respectively), those with a low BMI (< 18.5 kg/m^2^) (16.0%, 14.4%, and 13.4%, respectively), those with a low serum albumin level (< 3.5 g/dL) (26.6%, 25.0%, and 20.9%, respectively), and those requiring preoperative blood transfusion (4.1%, 2.9%, and 1.8%, respectively) than type B- and type A-certified institutions. Dependence in activities of daily living was also more frequently observed in patients at non-certified institutions than in those at type B- and type A-certified institutions (5.2%, 4.3%, and 2.6%, respectively). In contrast, preoperative chemotherapy was more common in type A-certified institutions (14.8%) than in type B-certified institutions (9.3%) and non-certified institutions (6.6%).

In terms of intraoperative findings, patients in non-certified institutions (277 min) had a shorter median surgical duration than those in type B-certified institutions (307 min) and type A-certified institutions (337 min). Meanwhile, patients in non-certified institutions had a greater estimated blood loss volume than those in type B- and type A-certified institutions (206, 153, and 130 mL, respectively). The use of MIS was lower in patients at non-certified institutions (29.2%) than in those at type B-certified institutions (44.9%) and type A-certified institutions (55.8%).

### Outcomes of total gastrectomy

In terms of postoperative outcomes, the rate of grade ≥ 3 complications based on the Clavien–Dindo classification system was slightly lower in non-certified institutions (9.3%) than in type B-certified (10.4%) and type A-certified (10.3%) institutions. However, non-certified institutions (2.2%) had the highest surgery-related mortality rate, followed by type B-certified (1.3%) and type A-certified (0.7%) institutions.

When evaluating the association between postoperative complications and surgery-related mortality and facility type after adjusting for the aforementioned covariates, the risk of postoperative complications was significantly higher among patients in both type B-certified institutions (risk-adjusted OR: 1.17, 95% CI: 1.03–1.33) and type A-certified institutions (risk-adjusted OR: 1.25, 95% CI: 1.09–1.44) compared with non-certified institutions. Nevertheless, the postoperative mortality risk was significantly lower in type B-certified institutions (risk-adjusted OR: 0.67, 95% CI: 0.51–0.88) and type A-certified institutions (risk-adjusted OR: 0.41, 95% CI: 0.29–0.58) (Table 4) than in non-certified institutions.

**Table 4:** Association between institutional certification status and (a) postoperative complications and (b) surgery-related mortality after total gastrectomy

(a)			
Facility-certified attributes	Unadjusted odds ratio (95% CI)	Risk adjusted odds ratio (95% CI)	*P* value
Non-certified institutions	References	References	
Type B-certified institutions	1.13 (1.02–1.25)	1.17 (1.03–1.33)	0.02
Type A-certified institutions	1.12 (1.01–1.25)	1.25 (1.09–1.44)	0.002

(b)			
Facility-certified attributes	Unadjusted odds ratio (95% CI)	Risk adjusted odds ratio (95% CI)	*P* value
Non-certified institutions	References	References	
Type B-certified institutions	0.59 (0.46–0.76)	0.67 (0.51–0.88)	0.004
Type A-certified institutions	0.31 (0.23–0.43)	0.41 (0.29–0.58)	< 0.001

CI: confidence interval

## Discussion

This nationwide study investigated the association between hospital certification status and postoperative outcomes in patients undergoing distal gastrectomy and total gastrectomy for gastric cancer using data from the NCD in Japan. For distal gastrectomy, the postoperative complication rates were 6.6%, 6.4%, and 5.7% at non-certified, Type B, and Type A certified institutions, respectively, which were not significantly different after multivariable adjustment. However, the mortality rates at these institutions were 1.6%, 0.8%, and 0.4%, which were statistically significant differences. For total gastrectomy, the complication rates were 9.3%, 10.4%, and 10.3%. Mortality rates were 2.2%, 1.3%, and 0.7%. Although multivariable analysis demonstrated a significant increase in complication rates for total gastrectomy at certified institutions, the absolute difference was small (approximately 1%) and its clinical relevance appears limited. In contrast, differences in mortality were both statistically and clinically substantial, suggesting that certified institutions are associated with reduced mortality.

Although detailed characteristics of postoperative complications and the underlying mechanisms of their occurrence—including the potential influence of patient background, preoperative treatment, and surgical or intraoperative factors—could not be fully evaluated in this study, we did identify several patient and treatment characteristics that differed between certified and non-certified institutions. Patients at certified institutions tended to be younger and have better nutritional status compared with those at non-certified institutions. For distal gastrectomy, certified institutions tended to treat fewer T3–T4 tumors and fewer cases with lymph node metastasis. For total gastrectomy, certified institutions treated a higher proportion of T3–T4 tumors, with little difference in the frequency of nodal involvement. Certified institutions also performed preoperative chemotherapy more frequently and had a higher proportion of MISs. These patterns were observed descriptively and likely reflect the characteristics of the patients and treatment strategies at certified institutions. Although the main analysis included risk adjustment for major preoperative factors, MIS was not included as an adjustment variable because it likely acts as an intermediate factor between certification status and surgical outcomes. Further analysis is necessary to clarify the relationship between the higher complication rates for total gastrectomy observed at certified institutions and factors such as a greater proportion of advanced disease and the more frequent use of technically demanding procedures, including complex MIS approaches.

Regarding mortality, certified institutions showed more favorable outcomes than non-certified ones even after adjusting for key patient-level risk factors. From an international perspective, postoperative mortality is considered a critical indicator of surgical quality. This interpretation aligns with the widely recognized concept of “failure to rescue (FTR)”, which emphasizes institutional ability to manage complications effectively once they occur [15, 16]. A previous nationwide report from the NCD [17] reported that the mortality rate of total gastrectomy is 2.2%, which is the same as that for non-certified institutions in the present study. Our study found that the mortality rates of total gastrectomy at Certified B and A institutions were 1.3% and 0.7%, respectively. As shown in the supplemental table, certification requires that board-certified gastroenterological surgeons and physicians are permanently assigned to the institution and holding regular multidisciplinary conferences. These requirements promote a well-organized, team-based approach to perioperative management, which likely contributes to the consistently lower mortality observed at certified institutions. This advantage may reflect effective case selection as well as differences in perioperative management, underscoring the clinical value of certification in ensuring institutional quality.

This large, nation-wide study presents the real world outcomes of the patients who underwent distal or total gastrectomy at certified and non-certified institutions. However, several limitations of this study must be acknowledged. First, detailed information on specific complications, as well as on the timing and causes of death, could not be collected, which limits mechanistic interpretation. Second, because NCD data do not include institutional- or intraoperative-level factors such as surgical complexity or details of postoperative management, causal pathways between certified institutions and outcomes could not be evaluated. Future studies utilizing more detailed data are warranted to clarify the mechanisms underlying complication and mortality differences and to examine complication-specific outcomes stratified by patient- and treatment-related factors.

In conclusion, this study compared short-term postoperative outcomes between certified and non-certified institutions. Although complication rates for distal gastrectomy did not differ significantly, certified institutions demonstrated slightly higher complication rates for total gastrectomy. This may reflect the situation in 2020–2022, when technically demanding procedures such as minimally invasive surgery—particularly for advanced or pretreated gastric cancer requiring total gastrectomy—were predominantly performed at centers of excellence. More importantly, postoperative mortality was consistently and substantially lower at certified institutions, highlighting their institutional capacity to cope with postoperative complications. These findings may reflect the superiority of multidisciplinary perioperative management at certified institutions.
